# Editorial: Structure, Function, and Evolution of E3 Ligases and Targets

**DOI:** 10.3389/fpls.2021.767281

**Published:** 2021-10-11

**Authors:** Derek J. Gingerich, Hanjo Hellmann, Matthew J. Christians, Sophia L. Stone

**Affiliations:** ^1^Department of Biology, University of Wisconsin-Eau Claire, Eau Claire, WI, United States; ^2^School of Biological Sciences, Washington State University, Pullman, WA, United States; ^3^Department of Cell and Molecular Biology, Grand Valley State University, Allendale, MI, United States; ^4^Department of Biology, Dalhousie University, Halifax, NS, Canada

**Keywords:** ubiquitin (Ub), E3 Ub-ligase, ubiquitin-like proteins (UBLs), ubiquitylation (Ubiquitination), ubiquitin 26S-proteasome system, E3-ligases

## Introduction

To respond to the environment and regulate growth and development, it is critical for organisms to precisely modulate the activity of proteins in their cells. One mechanism by which this regulation occurs is the covalent attachment of ubiquitin (Ub) and other ubiquitin-like proteins (UBLs) to specific target proteins. These modifications can alter the function, location, or levels of the target. Attachment of the majority of these modifiers occurs by an ATP-dependent E1/E2/E3 conjugation cascade during which a thioester linkage is formed between the Ub/UBL and the E1, followed by a transthiolation reaction where the Ub/UBL is transferred to the E2, then attachment of the Ub/UBL to the target protein via an isopeptide bond. This final step is typically mediated by E3 ligases, which recruit the E2, bind the substrate, and catalyze linkage between the Ub/UBL and the target.

## UBL E3-Ligases

**Figure 1:**
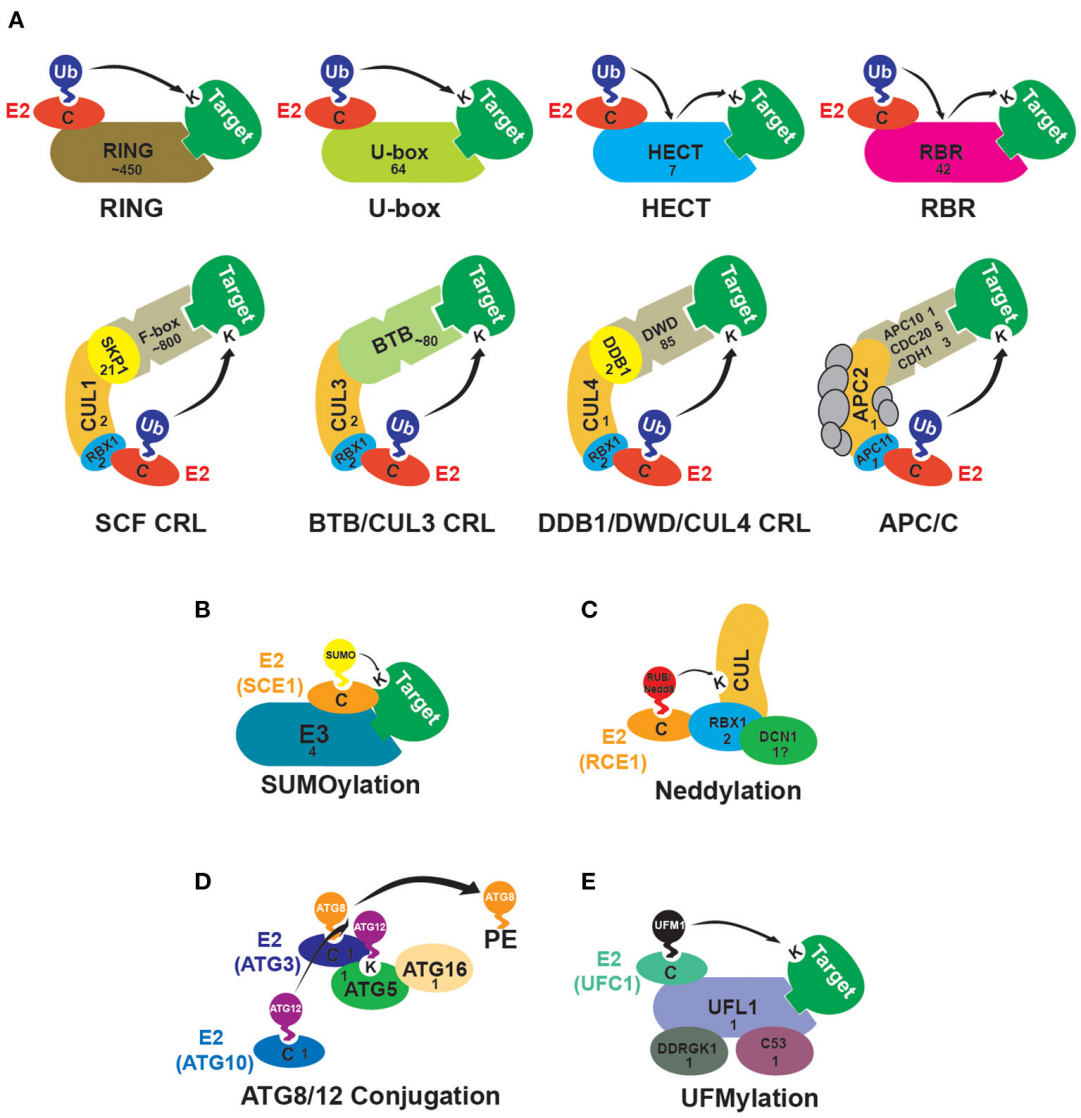
Subunit composition of E3-ligases in plants. The numbers displayed within each subunit indicate the number of genes in the *Arabidopsis thaliana* genome putatively encoding the E3 ligase or E3 ligase component. **(A)** Schematics of E3 ubiquitin (Ub)-ligases in plants. RING (*R*eally-*I*nteresting *N*ew *G*ene) and U-box E3 ligases bind an E2 and the target and facilitate direct transfer of Ub to the target protein. HECT (*H*omologous to the *E*6AP *C*arboxyl *T*erminus) and RBR (*R*ING-in-*B*etween-*R*ING) E3 ligases transfer the Ub from the E2 to a cysteine residue in the E3, then from the cysteine to the target protein. The CRLs (*C*ullin-*R*ING *L*igases) are multi-subunit structures with a Cullin (CUL) backbone protein that binds the E2 and a target adapter protein/complex. In SCF (*S*kp1/*C*UL1/*F*-box) complexes target adaptor F-box proteins bind to an Skp1 (*S*-phase *K*inase-associated *P*rotein 1)-like protein which interacts with CUL1. In the CRL3 complexes, a BTB (*B*road-Complex, *T*ramtrack, and *B*ric-à-Brac) domain-containing protein binds to the target and CUL3. In the CRL4 complexes, WD40 domain-containing DWD (*D*DB1 Binding *WD*40) proteins bind the targets and interact with CUL4 through a DDB1 (*D*amaged *D*NA *B*inding 1) protein bridge. The APC/C (*A*naphase-*P*romoting *C*omplex/*C*yclosome) contains at least 11 subunits. APC2 is a Cullin-like protein and APC11 is similar to RBX1 (*R*ing *B*o*x* Protein 1). Target recognition occurs via the CDC20 (*C*ell *D*ivision *C*ycle 20), CDH1 (*CD*C20 *H*omolog 1), and APC10 subunits (Gray et al., 2002; Lechner et al., 2002; Schroeder et al., 2002; Capron et al., 2003; Downes et al., 2003; Gingerich et al., 2005; Stone et al., 2005; Kong et al., 2007; Lee et al., 2008; Lima et al., 2010; Marín, 2010; Trujillo, 2018; Saleme et al., 2021). **(B)** Schematic of the SUMO (*S*mall *U*b-like *Mo*difier) E3-ligases. While SUMO E2s can directly interact with target proteins, the E3-ligases stimulate SUMO discharge by the E2 (Augustine and Vierstra, 2018) (https://www.frontiersin.org/articles/10.3389/fpls.2021.652170/full). **(C)** Proteins involved in the neddylation of Cullins. RBX1 functions synergistically with DCN1 (*D*efective in *C*ullin *N*eddylation 1) to facilitate the transfer of RUB (*R*elated to *Ub*iquitin)/Nedd8 from the E2 RCE1 (*R*UB-*C*onjugating *E*nzyme 1) to Cullins. There are three possible homologs of DCN1 in Arabidopsis, though two lack the UBA (*Ub*iquitin-*A*ssociated) domain seen in other DCN1 proteins (Merlet et al., 2009; Hosp et al., 2014). **(D)** Schematic of the ATG8 (*A*u*t*opha*g*y-Related 8)/ATG12 conjugation pathway. The UBL ATG12 forms a thioester linkage with the E2 enzyme ATG10 and then is conjugated to ATG5. The UBL ATG8 is transferred to the E2 enzyme ATG3, then the ATG5-ATG12 conjugate, together with ATG16, acts as an E3 facilitating ATG8-phosphatidylethanolamine (PE) conjugation (Doelling et al., 2002; Bassham et al., 2006; Mizushima, 2020). **(E)** UFM1 (*U*biquitin-*F*old *M*odifier 1) conjugation. UFL1 (*UF*M1-Specific *L*igase 1) is the E3-ligase for UFM1 and catalyzes the UFMylation of multiple substrates. UFL1 ligase activity requires interaction with an adaptor protein called DDRGK1 (*DDRGK* Domain-Containing Protein 1). Another UFL1 interactor, CDK5RAP3 (*CDK5 R*egulatory Subunit-*A*ssociated *P*rotein 3), affects the UFMylation profile and may serve as a substrate adaptor. DDRGK1 and CDK5RAP3 homologs are present in Arabidopsis; the CDK5RAP3 homolog is called C53 (Daniel and Liebau, 2014; Stephani et al., 2021; Witting and Mulder, 2021).

UBLs which use an E1/E2/E3-like reaction cascade for target attachment include SUMO (*S*mall *U*b-like *Mo*difier), RUB (*R*elated to *Ub*iquitin, called Nedd8 in yeast and animals), ATG8 (*A*u*t*opha*g*y-Related 8) and ATG12 (which are conjugated to the lipid phosphatidylethanolamine and the ATG5 protein, respectively), and UFM1 (*U*biquitin-*F*old *M*odifier 1) (Vierstra, 2012; Callis, 2014; Daniel and Liebau, 2014; Augustine and Vierstra, 2018; Witting and Mulder, 2021). The attachment of UBLs to targets is mediated by small families of E3-ligases (Figure 1). An example is SUMO and in this Research Topic Jmii and Cappadocia describe this interesting group of E3s involved in SUMOylation, which serves as a regulatory tool to change subcellular localization, complex function, or the protein-protein interactions of targets. Although plants encode very few SUMO E3-ligases, they broadly affect abiotic stress and hormonal responses, developmental processes, and cell-cycle regulation. Further insights into the structural organization and details on cellular processes are provided in the review (Jmii and Cappadocia).

## UB E3-Ligases

In contrast to the E3s involved in UBL conjugation, Ub-E3 ligases are a huge and diverse group. They divide into three main types: the RING (*R*eally-*I*nteresting *N*ew *G*ene) family, the HECT (*H*omologous to the *E*6AP *C*arboxyl *T*erminus) family, and the RBR (*R*ING-in-*B*etween-*R*ING) family (Downes et al., 2003; Hua and Vierstra, 2011; Callis, 2014; Dove and Klevit, 2017). The RING family divides into multiple subfamilies, including monomeric, dimeric, and heteromeric RINGs, the U-box type, the APC/C (*A*naphase-*P*romoting *C*omplex/*C*yclosome), and the Cullin-RING ligases (CRLs) (Figure 1) (Stone et al., 2005; Vierstra, 2009; Chen and Hellmann, 2013). Higher eukaryotes encode large numbers of Ub-E3 ligases in their genomes, and this is particularly so in plants (Grau-Bové et al., 2015). *Arabidopsis thaliana*, for instance, has ~1,500 loci encoding potential Ub E3s or E3 subunits (Figure 1). Many of these encode the subunits involved in target recognition. For instance, in Arabidopsis, there are ~800 genes encoding F-box proteins, the target adaptors in the SCF (*S*kp1/*C*UL1/*F*-box) CRL complexes (Hua et al., 2011; Hua, 2021), ~450 genes encoding the single-subunit RING E3s (Stone et al., 2005), and ~80 genes encoding the BTB (*B*road-Complex, *T*ramtrack, and *B*ric-à-Brac) protein target adaptors of the BTB/Cullin 3 CRL complexes (Gingerich et al., 2005) (Figure 1). Besides being large, plant E3 families have also undergone dynamic evolution, with diversification in family composition and sizes, and a high rate of gene gains and losses (Grau-Bové et al., 2015; Hua and Yu, 2019; Hua, 2021). This polymorphic nature complicates our ability to predict which family members are active or occur as pseudogenes and present challenges to researchers hoping to elucidate their roles. For these reasons, to date, the functions and targets of the majority of E3 substrate adaptors have not been determined. For instance, among the members of the relatively well-characterized Arabidopsis family of BTB proteins putative biological functions have been identified for only 39 (Christie et al., 2018; Ban and Estelle, 2021). For the F-box family, the percentage is even lower, with just 83 F-box protein-encoding genes (~10%) having a function identified via genetic characterization (Hua, 2021). With the majority uncharacterized, determining which are worth spending resources to investigate can be difficult. The development of better approaches to predict and separate active genes from pseudogenes would be crucial. In this Research Topic, Li et al. present such a method, using the F-box gene superfamily in Arabidopsis to demonstrate a novel neural network machine learning approach to classify genes as either functionally active or inactive. By integrating numerous features, their approach may be used to identify active gene candidates, allowing future work to focus on family members most likely to play roles in plant growth and development (Li et al.).

Ultimately, a full understanding of any individual E3-ligase requires dedicated study using a variety of approaches and here we present several articles that describe the characterization of individual E3s and/or their targets. Linden et al. used genetics to characterize the *Arabidopsis thaliana* RING E3 BRIZ, a heteromeric ligase that contains the BRIZ1 (*B*RAP2-*RI*NG-*Z*nf Domain 1) and BRIZ2 proteins. They show that *briz1* and *briz2* mutants fail to germinate or arrest early in seedling development and that phenotype is reduced when abscisic acid (ABA) levels are reduced. While the target(s) of BRIZ are still unknown, their data suggest that the function of the BRIZ ligase is to suppress ABA response early in development (Linden et al.) The research article from Beathard et al. focuses on a three members of the S23 subfamily of MYB transcription factors. These targets of the ubiquitin proteasome pathway are likely regulated by CUL3-dependent CRLs that use the BTB BPM (*B*TB/*P*OZ-*M*ATH) proteins as substrate adaptors. Genetic approaches, including overexpression and down-regulation via a polycistronic microRNA, implicate this MYB subfamily in stress and ABA response pathways, adding another facet to abiotic stress-regulation in context with the ubiquitin proteasome pathway (Beathard et al.). The RING E3 ligase COP1 (*Co*nstitutive *P*hotomorphogenesis 1) is a long-known master regulator controlling the switch from skoto- to photomorphogenesis in plants. The review article from Ponnu and Hoecker describes the regulatory steps that control COP1 activities, and nicely outlines this highly complex topic with insights into how this E3 ligase integrates different light qualities in concert with other proteins. In addition to discussing its role in other processes, such as temperature response, hormonal signaling, or cell division, a comparison between COP1 in plants and animals is provided (Ponnu and Hoecker). Finally, Erffelinck et al. describe the use of a yeast two-hybrid protein-protein interaction screen to identify the binding partners of the *Medicago truncatula* RING membrane-anchor-type E3 MKB1 (*M*A*K*I*B*ISHI1). MKB1 recruits the ER-associated degradation (ERAD) machinery to regulate levels of the enzyme HMGR (3-*H*ydroxy-3-*M*ethyl*g*lutaryl-CoA *R*eductase), which is a rate-limiting enzyme in the mevalonate pathway that supplies building blocks for the production of triterpene defense compounds. The researchers were able to identify an E2 enzyme for MKB1 and a heat shock protein that interacts with MKB1 and supports its activity. This work thus identifies additional key components of the MKB1-dependent ERAD machinery (Erffelinck et al.).

## Translating E3-Ligase Research to the Field

The articles in this Research Topic illustrate the wide range of developmental and environmental-response pathways in which E3-ligases are involved. Among these are pathways of agronomic significance, such as those that respond to abiotic stresses including drought, salinity, and temperature. Melo et al. provide a review of what is known about the roles of E3-ligases in abiotic stress responses in rice, with a focus on the monomeric RING and U-box E3s. In addition to providing a detailed description of individual E3s, they also summarize key areas for which more investigation is needed to better understand how these E3s function in these responses. Finally, they suggest stress response-related E3s could be targeted for genetic manipulation, allowing for the production of crops better suited for stress-prone environments (Melo et al.).

## Conclusions and Future Directions

In summary, this Research Topic presents significant contributions to understanding of E3-ligases. However, there is still much to be done to fully elucidate their functions. The targets of the majority of E3 ligases and the pathways in which they operate have yet to be determined. How E3/target interactions are regulated, the structures of the E3/target complexes, and how the E3s and the targets have co-evolved are all areas about which much more still needs to be learned. Increased understanding of these ligases may lead to new E3-centered strategies to modulate responses to biotic and abiotic stresses to improve crop agronomic performance. We anticipate that the study of E3-ligases and the Ub/Ubl conjugation pathways will continue to be a highly active, fruitful field, and we encourage more to join us in the study of these fascinating regulators.

## Author Contributions

DG was the primary author of this editorial. HH and MC provided summaries of articles that are used in the editorial and provided critical review of the editorial. SS provided critical review of the editorial. All four individuals were editors for the research topic.

## Conflict of Interest

The authors declare that the research was conducted in the absence of any commercial or financial relationships that could be construed as a potential conflict of interest.

## Publisher's Note

All claims expressed in this article are solely those of the authors and do not necessarily represent those of their affiliated organizations, or those of the publisher, the editors and the reviewers. Any product that may be evaluated in this article, or claim that may be made by its manufacturer, is not guaranteed or endorsed by the publisher.
